# Addressing Failures in Achieving Hypertension Control in Low- and Middle-Income Settings through Simplified Treatment Algorithms

**DOI:** 10.5334/gh.1082

**Published:** 2022-04-12

**Authors:** Jennifer Cohn, Helen Bygrave, Teri Roberts, Taskeen Khan, Dike Ojji, Pedro Ordunez

**Affiliations:** 1 Resolve to Save Lives, New York, US; 2 International AIDS Society, Geneva, CH; 3 Médecins Sans Frontierès Access Campaign, Geneva, CH; 4 Department of Public Health Medicine, University of Pretoria, Pretoria, ZA; 5 World Health Organization, Geneva, CH; 6 Department of Internal Medicine, Faculty of Clinical Sciences, College of Health Sciences, University of Abuja, Abuja, NG; 7 Department of Non-Communicable Diseases and Mental Health, Pan American Health Organization, Washington DC, US

**Keywords:** hypertension, treatment, health policy

## Abstract

Hypertension is the most important risk factor for cardiovascular diseases (CVDs), which are the leading global cause of death. Hypertension is under-diagnosed and under-treated in most low- and middle-income countries (LMICs). Current algorithms for hypertension treatment are complex for the healthcare worker, limit decentralization, complicate procurement and often translate to a large pill burden for the person with hypertension. We summarize evidence supporting implementation of simple, algorithmic, accessible, non-toxic and effective (SAANE) algorithms to provide a feasible way to access and maintain quality care for hypertension. Implementation of these algorithms will enable task shifting to less specialised health care workers and lay cadres, provision of fixed dose combinations, consolidation of the market while retaining generic competition, simplification of laboratory requirements, and lowering costs for health systems and people who incur out of pocket expenses.

## Introduction

Cardiovascular diseases (CVDs) are the leading cause of death globally, representing 31% of global mortality and causing 18.6 million deaths in 2019 [1,2]. Hypertension, a condition that affects approximately 1.4 billion people, is the single most important risk factor for CVD. Approximately 50% of heart disease, stroke, and heart failure cases are attributable to hypertension [3]. Further, the economic impact is huge, with an estimated 10% of global health care spending directly related to hypertension and its complications [4]. Hypertension often presents with no symptoms, earning it the title of ‘silent killer’ [5,6]. Effective treatment of blood pressure reduces stroke, heart disease and mortality, and investments in its treatment and control are cost-effective [7,8]. Unfortunately, hypertension is under-diagnosed and under-treated in most low- and middle-income countries (LMICs), where it is estimated that on average just 10% of people have achieved hypertension control [6].

One of the cornerstones of a successful public health approach for management across diseases is the use of standardized, evidence-based treatment algorithms. The progression of HIV treatment practices serves as an example. Initially, guidance from the World Health Organization (WHO) for the use of antiretroviral therapy (ART) listed eight triple combinations of antiretroviral (ARV) treatments as options for first line treatment [9]. Over time and through an optimisation approach, the WHO recommended one preferred first-line, hence simplifying procurement, supply, and clinical guidance. By contrast, standardized treatment algorithms for hypertension do not yet exist in many countries, thereby preventing simplification. At the global level, normative guidelines do not currently recommend a standard algorithm for hypertension. The HEARTS technical package, the WHO guidance for hypertension management, recommends nine possible algorithms, but does not endorse a preferred single algorithm or the preferred drug within any one class within an algorithm [10].

In this paper, we argue, from the point of view of key stakeholders, why implementation of a simple, algorithmic, accessible, non-toxic and effective (SAANE) treatment algorithm for hypertension can improve access to quality hypertension care, increase control rates and enable efficient, rational models of care and lower costs for medications. As the name suggests, SAANE algorithms are standardized, have few steps for clinicians to follow (simple, algorithmic), are composed of medicines that are available and affordable (accessible), have a good side effect profile (non-toxic/well-tolerated) and are clinically effective across most populations (effective). At a time when the COVID-19 pandemic is facilitating the fast-tracking of more efficient models of care world-over, the move to SAANE algorithms is more timely and urgent than ever.

## The current situation for global hypertension treatment

**Figure 1:**
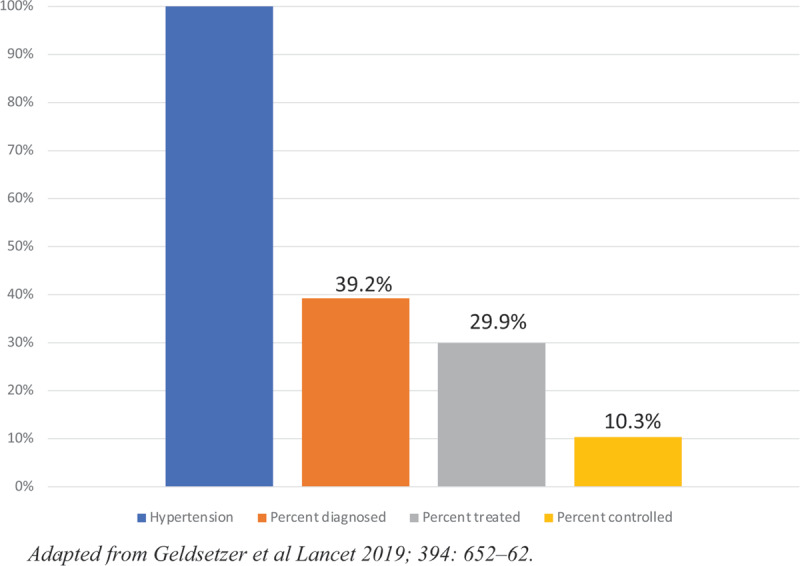
Cascade of hypertension control in low- and middle-income countries.

**Figure 2:**
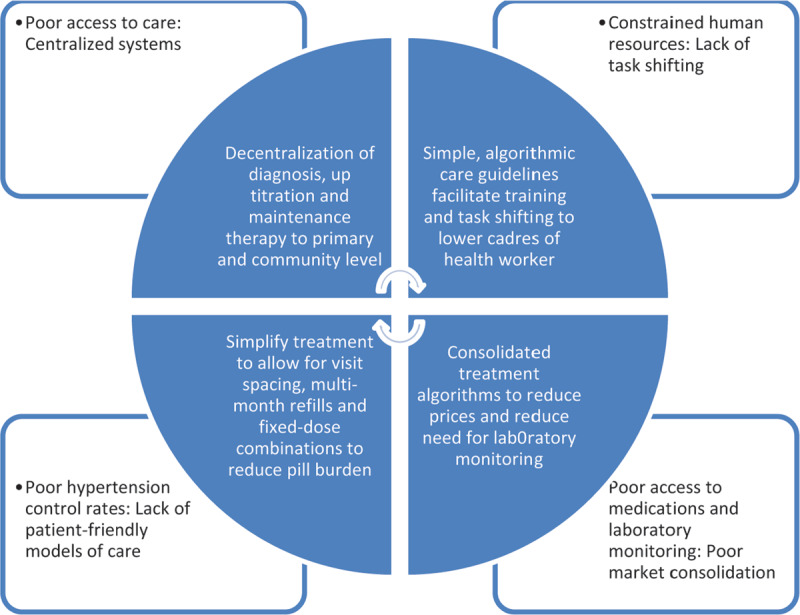
Barriers and enablers for sustainable, effective, and scalable hypertension programs.

Access to hypertension care is inadequate in most LMICs, resulting in a cascade of care characterized by inadequate diagnosis, treatment and control (***Figure 1***) [6]. A number of key barriers currently prevent scale-up of effective and sustainable hypertension treatment and care (***Figure 2***). Care for non-communicable diseases, such as hypertension, tends to be centralized with limited task-shifting to lower cadres of health workers, such as nurses, leading to poor access for patients and shortages of health workers able to provide adequate care [11,12]. Centralized, poorly accessible care, frequent appointments, significant out-of-pocket costs and high pill burden may also lead to poor control rates and high lost-to-follow-up. Further, although the market for hypertension medications is large and the cost of manufacture is low, there are large price disparities among countries, suggesting a non-optimized market and room for reduction in prices and streamlining of supply chains [13,14,15]. Scale-up of appropriate and accessible hypertension care is also hampered by budget constraints, with large gaps between demonstrated need for funding and real budget allocations [16,17]. Fortunately, the adoption of SAANE algorithms can enable the implementation of effective interventions to address these barriers. While additional investments will be required to scale up hypertension programs, SAANE algorithms and the models of care they enable can reduce the budget needed to scale up hypertension care in several areas, including medication prices, laboratory monitoring costs, and health system expenditures [18].

## Why simple treatment algorithms are needed by the health system

The sheer scale of the hypertension epidemic demands that we re-examine the service delivery models we currently provide for hypertension. Management of hypertension at the primary care level has been recommended through the WHO Package of Essential Interventions (PEN) package and the WHO HEARTS technical package recommends a team-based approach [19]. SAANE algorithms may enable this approach, reduce the average time needed to control blood pressure and improve overall control rates. Once blood pressure is controlled, people may then be considered eligible for less intensive follow-up. Differentiated service delivery (DSD), a concept recognised in the international HIV community, is a person-centred approach that simplifies and adapts HIV services across the cascade, in ways that both serve the needs of patients and reduces unnecessary burdens on the health system [20]. If SAANE algorithms improve blood pressure control rates, a larger proportion of people with hypertension could be managed with less frequent visits, be given multi-month refills and have community-based distribution of medication refills, hence reducing the number of people to be seen at facility level and reducing the burden on the health work force. Such an approach may also have cost efficiencies for the health system, as has been demonstrated by such models used to deliver lifelong ART [21].

### Health care workers

International and national hypertension guidelines are complex, involving multiple steps to initiate treatment, increase medication dose and add additional drug classes to achieve hypertension control. In 2018, the WHO published the HEARTS technical package [18], which provided nine possible algorithms that national programmes could consider for adoption. These algorithms indicate clear titration steps but do not go as far as selecting a preferred algorithm or naming the preferred drug within a class. Health workers, both physicians and non-physicians, especially those working in primary care, need SAANE algorithms in order to deliver universal and quality, person-centred care, and to achieve improved hypertension control.

SAANE algorithms facilitate task-sharing of hypertension management to lower cadres and decentralisation of care by streamlining and standardizing health worker trainings and reducing the need for complex clinical decision-making. Less specialized health workers, who work in more decentralized or remote clinics, can thus successfully initiate, titrate and maintain hypertension treatment with little oversight from more highly trained health workers. Such algorithms may enable task-sharing to trained and supervised community cadres. Several studies have suggested that task shifting to nurses for management in LMICs is feasible and potentially scalable, with one study demonstrating significant increases in skills and knowledge with only four training sessions [22,23]. In HIV task shifting to nurses for initiation and management of HIV treatment has been widely adopted and has resulted in up to 66% reduction in government expenditures for HIV follow-up care [24]. A systematic review of task shifting in LMICs across various disease areas has demonstrated that task shifting is both feasible and cost saving in areas like HIV, TB and has the potential for cost savings in non-communicable diseases like hypertension [25]. Investment in the development of training and mentorship of less specialized health workers as well as research to assess the feasibility and effectiveness of use of SAANE algorithms by such cadres is needed in a variety of settings. Finally, SAANE algorithms allow programme managers to assess the extent to which health workers are following treatment algorithms and enable both quality assessment and quality improvement initiatives.

SAANE algorithms facilitate not only the initiation of therapy, but also adjustment to reach hypertension control. Despite evidence that suggests that the majority of people with hypertension will require two drugs or more to achieve optimal and sustained control [26], reports have shown that most people only receive monotherapy [27]. The concept of clinical inertia has been described in the literature [28], highlighting the challenges for clinicians to intensify treatment, particularly where multiple treatment steps may be involved. This may be an additional challenge where task sharing to lower cadres is needed to meet demand. Including the use of rational fixed dose combinations (FDCs) in any step of a SAANE algorithm can simplify titration and help to overcome inertia. Overall, the use of clear algorithms has the potential to achieve control earlier, reducing the number of clinic visits needed to reach control, and therefore reducing the burden on the health system [5,29].

### Procurement and supply systems

Similar to the ART market, SAANE algorithms can improve quality and access to anti-hypertensive treatments by reducing prices [30], ensuring consistent supply by simplifying supply chain considerations, and enabling decentralized drug distribution to the community level. Multiple studies have demonstrated that anti-hypertensive medications are not affordable and available in LMICs, with costs of a month’s supply of a single anti-hypertensive medication in the public sector exceeding 10% of a typical government worker’s salary [13,14]. Availability of medications at the facility level is closely linked to both affordability and strength of the supply chain system. The availability of medications to treat CVD, including anti-hypertensive medication, is poor in LMICs, with recent studies suggesting only 7–26% availability of basic CVD medications in the public sector [15,31].

Despite the potentially large market for anti-hypertensive medications, the market is not optimized. Given the burden of hypertension in high-income countries, pharmaceutical companies have prioritized this sector, resulting in a large number of medications across the main anti-hypertensive classes. As many of the patents on anti-hypertensive drugs have expired, or were not filed in LMICs, each given molecule has the potential to have a wide number of competitive generic manufacturers. While competition is one of the most important factors in a given medication’s price, such a fragmented market may serve to fracture demand across a large number of products, thus diminishing the purchaser’s power to negotiate lower prices and, in some cases, reducing suppliers’ economies of scale, which increases production costs [32]. SAANE algorithms that focus on a limited number of molecules and related rational FDCs can help to consolidate the market while retaining competition. National procurement agencies or regional procurement bodies can then pool demand for preferred molecules and FDCs, increasing buying power and facilitating potential manufacturing efficiencies. Procurement should be focused on medications that are quality-assured and in countries with weak or developing national regulatory authorities, this may mean selecting medicines with approval from a stringent regulatory authority or other national regulatory authority with WHO maturity level 3 or 4.

SAANE algorithms will also simplify and facilitate more accurate and long-term forecasting of product needs both at local and national level. Forecasting based on longer-term orders or communication of more reliable product needs to manufacturers can facilitate manufacturer planning and increase the likelihood of manufacturers fulfilling orders on time and in full. The use of FDCs in any step of the SAANE algorithm can further simplify procurement and supply chain considerations. FDCs have the potential to minimize strain on under-resourced supply chains by reducing the number of products and related transactions in a given supply chain. In addition, the smaller packaging footprint of FDCs versus their individual components may reduce costs related to freight and decrease storage space needed for products at warehouse- and facility-level. Finally, use of SAANE algorithms, especially those that contain FDCs in any step, will allow for improvement in last-mile and community-delivery of medications as a more limited number of products with a greater amount of buffer per product will be delivered to each facility or for distribution at the community level. The result of the above efficiencies will be lower anti-hypertensive drug prices and improved availability.

### Laboratory systems

With some notable exceptions, such as rapid diagnostic tests for HIV and malaria, few tests are widely accessible in LMICs. As such, essential diagnostic packages have been based on the minimal requirements for diagnosis and treatment monitoring that cannot rely solely upon clinical signs and symptoms. Rationalizing the choice of drug therapy may also serve to simplify diagnostic choice, supply, reduce costs, and facilitate programmatic simplification and decentralization.

While it is recommended that laboratory monitoring for renal toxicity is available to people on hypertension treatment, algorithms must be based on minimal requirements to reduce treatment-related adverse events so as not to impede access to life-saving treatment in LMICs. The SAANE algorithm that reduces or eliminates the need for monitoring in the initial steps can reduce the need for laboratory monitoring and use of a standardized algorithm will allow for the identification of the minimal package of laboratory monitoring required for quality care [33].

There is a perceived lack of evidence on how to reduce laboratory monitoring requirements. However, much data exists already that shows no increase in serious adverse effects without laboratory monitoring and that very few people require more individualised approaches. For example, the SCREAM study developed a hyperkalaemia susceptibility score to decrease the need for regular monitoring of creatinine for those on ACE-I and ARB [34]. This was based on data that hyperkalaemia was rare among people with a GFR >60 mL/min per 1.73 m^2^ within the first year of treatment. Conversely, diuretics are more associated with hypokalaemia [35,36,37].

Unsurprisingly, many examples of diagnostic simplification have come from the HIV community, who used SAANE algorithms to determine minimal laboratory requirements to feasibly scale-up large and decentralised chronic disease programmes in LMICs and HIV-endemic settings. When tenofovir disoproxil fumarate (TDF) was first introduced as part of the preferred first-line regimen, the low risk of renal toxicity associated with the drug led the WHO to recommend creatinine testing before treatment initiation and for treatment monitoring [38]. However, given the lack of access to creatinine testing in LMICs, they added that it was not mandatory for initiating ART. A stronger recommendation was reserved for those at risk, e.g. with impaired GFR or underlying kidney disease, with the recommendation that kidney function could be adequately measured using a urine dipstick to detect renal toxicity rather than more complex laboratory-based tests for creatinine clearance.

In the absence of sufficient clinical evidence, or cohort data from a variety of settings, including low resource settings, modelling could help fill an evidence gap to increase the confidence in simplified laboratory approaches. For renin-angiotensin system inhibitors, the added value of regular creatinine monitoring could be modelled to calculate the cost versus benefit of the clinical risk versus no or a low frequency of monitoring.

## Why simple treatment algorithms are needed for people living with hypertension

People with hypertension need SAANE algorithms for effective and faster blood pressure control and to reduce their morbidity and mortality. Simple treatment algorithms have been shown to significantly improve control rates for a variety of diseases ranging from hypertension to HIV and TB in LMICs. In one study in China, implementing standard hypertension treatment algorithms increased treatment by 64% and control by 98% [39]. The rapid scale up of global HIV treatment was based on a public health approach using SAANE protocols, with 27.5 million people on HIV treatment by the end of 2020 and the majority of high-burden low- and middle-income countries recommending the same first and second-line treatments [40]. Further, this public health approach has resulted in 96% treatment success within one year in both South Africa under a public health approach using SAANE algorithms and in Switzerland under an individualized, resource-intense treatment approach [41]. SAANE protocols may also facilitate hypertension control by improving the quality and person-centredness of services delivered by reducing pill burden, frequent facility visits and waiting times.

As an enabler of DSD, SAANE algorithms may improve retention in care and sustain hypertension control [42], reducing long-term complications of hypertension and, ultimately, mortality [43]. The benefits of such DSD models have been demonstrated in ART delivery, reducing both transport and opportunity costs for people and often reducing waiting times [44,45,46] when clinic visits are required – also a likely benefit of SAANE algorithms for hypertension. The provision of care at decentralized facilities by less specialized health workers also brings care closer to the person’s home, reducing their out-of-pocket transport costs and time off work or away from family responsibilities. Lastly, SAANE algorithms enable positive market forces to reduce medication costs, which is critical for people where medications are paid for out-of-pocket.

Current algorithms are not only complex for the healthcare worker but translate to a large pill burden for the person with hypertension. Hypertension treatment is lifelong, and adherence is a challenge. Interventions that reduce pill burden, including through the use of FDCs, have the potential to reduce the barriers to maintaining lifelong treatment and improve both the quality of life for the person with hypertension and the clinical outcomes of the treatment [47].

## Why simple treatment algorithms are needed by domestic funders and international donors

Programmes supporting non-communicable diseases such as hypertension in LMICs are characterized by constrained budgets and the need to maximize efficiencies [48]. SAANE algorithms have the potential to reduce costs at several levels. Procurers, including national programmes and donor organisations, can focus market-shaping interventions on demand for quality-assured priority products, including rational FDCs, through volume commitments. A limited number of medications and FDCs will allow governments and insurance providers to prioritize selection of products for essential medication lists, insurance formularies and public payments. Finally, support for SAANE algorithms enables better assessment of quality care, enabling quality improvement measures. With a clear SAANE algorithm, a programme can more easily assess if therapeutic inertia is significantly contributing to a lack of hypertension control among those on treatment.

## Conclusion

SAANE algorithms have great potential to enable hypertension programs to reach their full potential to reduce the CVD burden globally. The next step requires broad implementation and scale-up at programmatic level. This effort can begin with WHO’s leadership whereby guidance can be based on the review of both the clinical evidence and the need for a public health approach and programmatic aspects that facilitate feasibility. The initial step should be the selection of a short anti-hypertensive formulary or a core set of medications based on the presence of ideal characteristics from each of the main four groups of anti-hypertensive medications, followed by development of a preferred algorithm, with named classes, drugs and dosages. Thereafter, countries need to lead the way in programmatic implementation, along with documenting and sharing lessons learnt for successful approaches and build the evidence-base to support implementation elsewhere. Lastly, domestic and international resources must be allocated to ensure scalable and sustainable hypertension programmes globally.
